# Front-of-Pack Labeling in Chile: Effects on Employment, Real Wages, and Firms’ Profits after Three Years of Its Implementation

**DOI:** 10.3390/nu14020295

**Published:** 2022-01-11

**Authors:** Guillermo Paraje, Daniela Montes de Oca, Juan Marcos Wlasiuk, Mario Canales, Barry M. Popkin

**Affiliations:** 1 Business School, Universidad Adolfo Ibáñez, Avenida Diagonal Las Torres 2640, Peñalolén, Santiago 7910000, Chile; guillermo.paraje@uai.cl; 2 Instituto de Nutrición y Tecnología de los Alimentos (INTA), Universidad de Chile, Macul 7810000, Chile; dmontesdeoca@alumnosuaicl.onmicrosoft.com; 3 Central Bank of Chile, Agustinas 1180, Santiago 8320000, Chile; jwlasiuk@bcentral.cl (J.M.W.); mcanales@bcentral.cl (M.C.); 4 Department of Nutrition, University of North Carolina at Chapel Hill, 123 W Franklin St, Suite 210, CB 8120, Chapel Hill, NC 27516, USA

**Keywords:** Chile, food and beverage labeling, employment, wages, profits

## Abstract

This study evaluates the impact of Chile’s innovative law on Food Labeling and Advertising, enacted in June 2016, on employment and real wages and profit margins for the food and beverage manufacturing sectors in the 2016–2019 period, using unique company-specific monthly data from Chile’s tax collection agency (measuring aggregate employment, real wages, average size of firms, and gross profit margins of the food and beverage manufacturing sector). Interrupted-time series analyses (ITSA) on administrative data from tax-paying firms was used and compared to synthetic control groups of sectors not affected by the regulations. ITSA results show no effect on aggregate employment nor on the average size of the firms, while they show negligible effects on real wages and gross margin of profits (as proportion of total sales), after the first two stages of the implementation (36 months), despite significant decreases in consumption in certain categories (sugar-sweetened beverages, breakfast cereals, etc.). Despite the large declines found in purchases of unhealthy foods, employment did not change and impacts on other economic outcomes were small. Though Chile’s law, is peculiar there is no reason to believe that if similar regulations were adopted elsewhere, they would have different results.

## 1. Introduction

Chile developed a coordinated comprehensive set of policies to significantly reduce ultra-processed food (UPF) consumption and a nutrient profile model to unite all policies [1,2]. Planners first created the model to delineate products with excessive nutrient content, or that were high in added sugar, added saturated fat, or added sodium, and whether any of those additions contributed to higher energy density of foods [3]. Chile was the first country to implement a mandatory national front-of-pack (FOP) nutrient warning label policy, followed by Peru, Uruguay, Israel, Mexico, and Argentina. Limits of critical nutrients were decreased gradually to allow producers to adjust their production processes (as shown in Appendix A, Table A1).

Chile’s food marketing policies are worth special consideration as these are the most comprehensive of any country to date [4]. The law also prohibits marketing high-in (sugar, fat, sodium) products to children under 14 years of age or marketing with themes or promotional strategies that appeal to children regardless of audience, media, or location. The prohibited marketing approaches include fun or fantasy themes, cartoon characters, animation, children’s music, child actors, situations representing children’s daily lives, free gifts or toys, contests, interactive games, or apps to attract children’s attention; the ban was later expanded to include any type of marketing from 6 am to 10 pm of high-in products.

**Figure 1:**
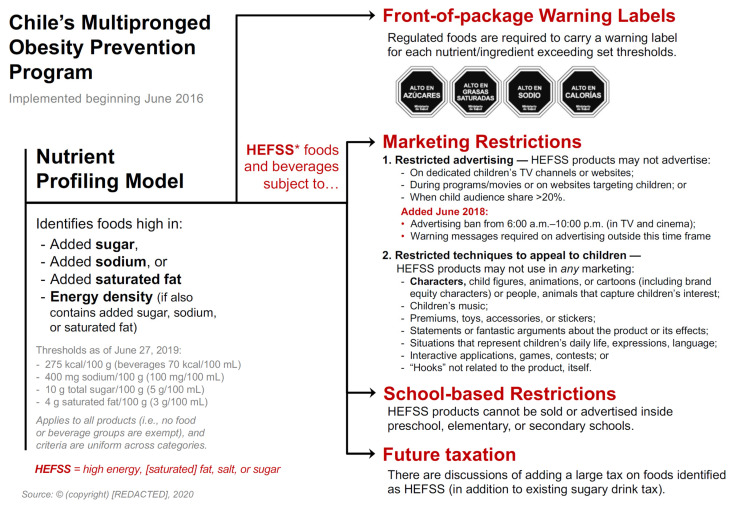
Chile’s multipronged Obesity Prevention Program. * HEFSS: high energy, (saturated) fat, salt, or sugar.

The overall law is laid out in Figure 1. Here we briefly summarize some results:

(a) Focus groups with low- and middle-income mothers suggest that after initial implementation of the law, profound changes in attitudes were found toward food purchases driven both by the knowledge mothers gained from these labels and by children telling their mothers not to purchase products with warning labels [3,5].
(b) Among high-in purchases (i.e., purchases in products with at least one front-of-package warning label), relative to the counterfactual statistic, there were notable relative declines of 23.8% of calories purchased, 36.7% of sodium purchased, and 26.7% of sugar purchased. There were larger absolute reductions in sugar from high-in beverages and larger reductions in calories, saturated fat, and sodium from high-in foods [6].
(c) An evaluation comparing the nutritional profiles of products before and after the first year of Chile’s FOP law found significant reductions in the proportion of products required to carry warning labels, suggesting that companies reformulated products to improve their health profiles and avoid the FOP warning label requirement [7].
(d) Research shows that the percentage of TV ads for foods high in energy, saturated fat, sugar, or sodium decreased from 41.9% before the regulation to 14.8% after the regulation’s implementation, resulting in a 44% decrease in exposure to “high-in” foods advertisement in children and a 58.0% decline for adolescents during the first year of the law implementation [8]. Total ads, however, did not change as they were shifted in the first phase to nonchild TV shows [9].

Lobby against the Law, which continues until today, was mostly based on the effects it would have on the food and beverage industry, particularly employment. Pro-industry think-tanks claimed that such regulations would affect profits and employment in the food sector [10]. These claims are often found when similar food labeling initiatives are discussed in Latin America (e.g., Mexico, Colombia, Brazil, Peru, Uruguay, and Argentina), Asia (India), and sub-Saharan Africa (South Africa) [11,12]. Arguments are based on simplistic textbook economic models with little or no empirical support.

A recent study conducted for Chile found that after 18 months of implementation of the FOP regulations (first stage of the implementation of FOP regulations started on the 26 June 2016) there was no discernible effect on employment nor on real wages, when comparing food industries likely affected by regulations with those not affected by them [9].

The purpose of this study is to build upon this evidence and to assess the impact of both the first (July 2016) and second stages (July 2018 to June 2019) of the implementation of Chile’s Law 20606 on the aggregate employment, the real wages, the average size of firms and the gross margin of profits of the food and beverage manufacturing sector. Using interrupted-time series analyses (ITSA) on administrative data from tax-paying firms, assessment was carried out on whether FOP regulations had a differential impact on food and beverage sectors when compared to synthetic control groups of sectors not affected by the regulations.

## 2. Materials and Methods

### 2.1. Data

Administrative tax records from Chile’s tax collection agency (the Servicio de Impuestos Internos -SII-) from January 2013 to May 2019 at the class level of the SII classification of economic activities were used for this study [13]. The SII collects monthly information for all firms in the formal sector of the economy (formal firms are those subjected to the FOP regulations) on number of employees, nominal wages, total nominal sales and nominal cost of raw materials and capital goods. Nominal wages include base salary, incentive pay, bonuses, employer-provided benefits, overtime pay, and any other included taxed item. Informal non-tax paying firms are not included in the database though there is evidence that the informal sector is not large in Chile (at least for Latin American standards). The Chilean National Statistics Institute (Instituto Nacional de Estadísticas -INE-) measured informal employment at the national level at 30.0% for the last quarter of 2019, compared with 28.8% for the manufacturing sector [14].

The SII classifies firms’ economic activities according to the class of activities firms declare to have using an ad-hoc classification system [13]. From this system, classes of economic activities related to the manufacturing of food and beverages can be clearly identified, as shown in Appendix A, Table A2.

The monthly average for the number of firms in the food and beverage manufacturing sector filing to the SII in the pre-intervention period (January 2013–June 2016) was 19,288; while such a figure was 22,519 and 26,159 for the first-intervention period (July 2016–June 2018) and second-intervention period (July 2018–June 2019), respectively. On the other hand, the average number of firms for the entire manufacturing sector (excluding the food and beverage manufacturing sector) filing to the SII in the pre-intervention period was 28,264; while such a figure was 30,339 and 33,933 for the first and second-intervention period, respectively.

### 2.2. Methods

The group of interest in the analyses is the entire food and beverage manufacturing sector. Some firms within this group were affected by the FOP regulations in different ways, while others were not affected at all. Affected firms may produce goods that are labeled and may also produce their unlabeled substitutes or might have had produced regulated goods that were subsequently reformulated. The data does not provide information on the proportion of sales within each firm that are labeled, or on the proportion of workers allocated to the production of each type of product (labeled vs. unlabeled). Focusing on the evolution of the entire food and beverage manufacturing sector allows for consideration of the effects of adjustments within firms for all firms and across them. If, due to the FOP regulations, certain firms within the food and beverage sector decrease, for instance, employment that is gained by other firms within the same sector, it is reasonable to say that the effect of FOP regulations on employment in that sector is neutral. Hence, variables of analyses are defined for the food and beverage manufacturing sector and for the group of industries outside such a sector that were not affected by FOP regulations and are used as control groups.

A monthly gross profit margin for the food and beverage manufacturing sector (or the control group) is defined as the difference of total monthly sales and the sum of monthly total wages and monthly costs of raw materials and capital goods, as a proportion of monthly total sales, for the food and beverage manufacturing sector (and the control group). Similarly, monthly aggregate employment for such a sector is defined as the sum of monthly employment for each of the classes of economic activities shown in Appendix A, Table A2 (or the control group). Average monthly employment by firm is defined as the ratio between monthly aggregate employment in the food and beverage manufacturing sector (or the control group) and the monthly number of firms in such a sector (or the control group).

Average monthly wages in the food and beverage manufacturing sector (or the control group), ${\bar{W}}_{t}$, are defined as

(1) $${\bar{W}}_{t}=\frac{\sum_{i=1}^{I}\left({\bar{w}}_{it}\times AE_{it}\right)}{AE_{t}}\;$$

where ${\bar{w}}_{it}$ is the average monthly gross wage for the ***i*** classes of economic activities shown in Appendix A, Table A2 (or the control group) at month ***t***; and $AE_{it}$ is aggregate employment for the ***i*** classes of economic activities shown in Appendix A, Table A2 (or the control group) at month ***t***. Nominal variables (total sales, wages, cost on raw materials, and capital goods) are deflated using the Consumer Price Index and expressed in constant pesos of December 2019 [15].

The effect of FOP regulations on the described variables is estimated using interrupted-time series analyses (ITSA) models with synthetic control groups, with two interventions. The first intervention was in July 2016, when the FOP regulations were first implemented, while the second intervention was in July 2018 when the second stage of the labeling was implemented (decreasing the limits of the nutrients, as shown in Appendix A, Table A1). ITSA allows to test differential changes in levels and trends between the treated group (food and beverage manufacturing sector) and the control group (classes of economic activities in sectors not affected at any time by the FOP regulations) [16]. It allows for testing the suitability of the chosen control group by comparing the pre-intervention trends and levels of this group with respect to the treated group. Suitable control groups allow for causal inferences to be made about the intervention, as they can replicate the statistical behavior of the treated group in the pre-intervention period. Any departures in behavior between treated and control groups after interventions, can be attributed to them.

The estimated equation is

(2) $$Y_{t}=\beta_{0}+\beta_{1}T_{t}+\beta_{2}X_{1t}+\beta_{3}X_{1t}T_{1t}+\beta_{4}Z+\beta_{5}ZT_{t}+\beta_{6}ZX_{1t}+\beta_{7}ZX_{1t}T_{1t}+\;\beta_{8}X_{2t}+\beta_{9}X_{2t}T_{2t}+\beta_{10}ZX_{2t}+\beta_{11}ZX_{2t}T_{2t}+\sum_{j=1}^{11}\delta_{j}D_{jt}+\gamma ln\left(IMACEC_{t}\right)+\epsilon_{t}\;$$

where ***Y_t_*** is the dependent variable (i.e., aggregate employment, average real wages, average employment, gross margin of profits, or number of firms) at time ***t***. In the case of aggregate employment, average employment per firm, and wages, their natural logarithm is used. ***X_it_*** is a categorical variable identifying the treatment period (where ***i*** = 1 if between July 2016 and June 2018, and ***i*** = 2 from July 2018 to June 2019); ***Z*** is a dichotomous variable identifying the treated group (0 if control group); ***T_it_*** are trend variables (***i*** = 1 is the trend since the start of the first intervention period; and ***i*** = 2 is a trend since the start of the second intervention period); and ***D_j_*** is a set of dichotomous variables for calendar months (to adjust for seasonality). We considered dummy variables accounting for an outlier month (July 2015) and for a tax change affecting sugar-sweetened beverages (SSB) (October 2014) but finally dropped them because of a lack of statistical significance in all models. IMACEC***_t_*** is the not seasonally adjusted non-mining Monthly Economic Activity Index (Índice Mensual de Actividad Económica, IMACEC) produced by the Central Bank of Chile, and included as a proxy for aggregate economic activity [17].

Two additional dependent variables were also considered, with results presented in the Appendix A, Table A3: the monthly number of firms and an alternative definition for the gross margin of profits defined as the difference of total monthly sales and the sum of monthly total wages and monthly costs of raw materials, as a proportion of monthly total sales, for the food and beverage manufacturing sector (or the control group).

Though all parameters of Equation (2) are important, some of them are of special interest [16,18]. First, parameters $\beta_{4}$ and $\beta_{5}$ represent the difference in level and trends of treated and control groups before the treatment, while $\beta_{6}$ and $\beta_{7}$ are the difference in the level and the trends between both groups in the period immediately following treatment, respectively. Non-significant $\beta_{4}$ and $\beta_{5}$ parameters imply that pre-intervention control and treatment groups have comparable statistical behaviors, while non-significant $\beta_{6}$ and $\beta_{7}$ parameters imply that the level and the trends of both groups, respectively, do not change after the first intervention.

Second, parameters $\beta_{10}$ and $\beta_{11}$ represent the difference in the level and the trends of treated and control groups between the first and the second intervention. Non-significant parameters imply that the second intervention do not differentially change levels or trends between treated and control groups after the second intervention, when compared to the first intervention, respectively.

It could be argued that employment, wages, and profits were mostly affected by the first intervention (July 2016), as at that time, firms went from facing no marketing regulations to facing all the regulations included in Law 20606. Following this argument, it could be argued that the second intervention (July 2018), which decreased the limits of the nutrients (as shown in Appendix A, Table A1) was a relatively minor change compared to the first one. To test this hypothesis, models such as the one shown in Equation (2) are estimated for the entire period (January 2013–June 2019) but consider only the first intervention (July 2016). In such a case, parameters $\beta_{8}$ to $\beta_{11}$ are set equal to zero. Thus, non-significant $\beta_{6}$ and $\beta_{7}$ parameters in these models imply FOP regulations implemented in July 2016 had no effect neither on the levels nor on the trends of treated groups with respect to control groups, when comparing to pre-intervention levels and trends.

A synthetic control group is an ad hoc group that relies on the weighing of observations from non-affected classes of economic activities that can mimic the statistical behavior (in levels and trends) of employment, wages, and profits of the affected sectors in the pre-intervention period (January 2013–June 2016). Hence, if after the first intervention levels and trends of the synthetic group are changed with respect to the treated group, such changes can be attributed to the intervention [19]. Because there is not a single class of economic activity capable of replicating the pre-intervention behavior of the treated group, the synthetic group is constructed using several “donors” (i.e., classes of economic activities not affected by the interventions) that are weighted using the SCUL (Synthetic Control Using Lasso) methodology [20]. As the name suggests, it applies *lasso* regressions, which are “penalized Ordinary Least Square (OLS)” to prevent the over-adjustment that a regular OLS would have in the usual presence of autocorrelation in time series, which would lead to inadequate predictions off-sample [20]. Details of the methodology and a package in R for its application are available elsewhere [20].

In the context of assessment of the FOP regulations, “donors” are selected from sectors or classes of economic activities that were not affected directly by those regulations, such as those in the non-metallic manufacturing sector, excluding food and beverage (e.g., manufacturing of textiles, chemics, plastics, paper, etc.). In case that no suitable donors are found in the non-metallic manufacturing sector, the search is extended to the metallic manufacturing sector (e.g., manufacturing of iron, copper, tools, car parts, etc.).

We estimated all the models in Stata 17 using the “itsa” command, which estimates linear interrupted-time series models using the ordinary least squares method with Newey–West robust standard error. We considered different lags to correct for autocorrelated errors after running Cumby–Huizinga general tests for autocorrelation [16].

## 3. Results

Table 1 shows descriptive statistics for the dependent variables for the treated (food and beverage manufacturing sectors) and synthetic control groups, for the pre-intervention period, and for periods after the first and second intervention. As can be seen in the pre-intervention period, the treated and control groups show similar means for aggregate employment, average (per worker) real wage, average number of workers per firm, and the gross margin of profits (as a proportion of sales). Aggregate employment in the food and beverage sector increased from an average of 184,971 workers in the pre-intervention period to an average of 209,663 workers during the second intervention period (an increase of 13.3%). Average real wages also increased between these two periods (an increase of 6.2%).

**Figure 2:**
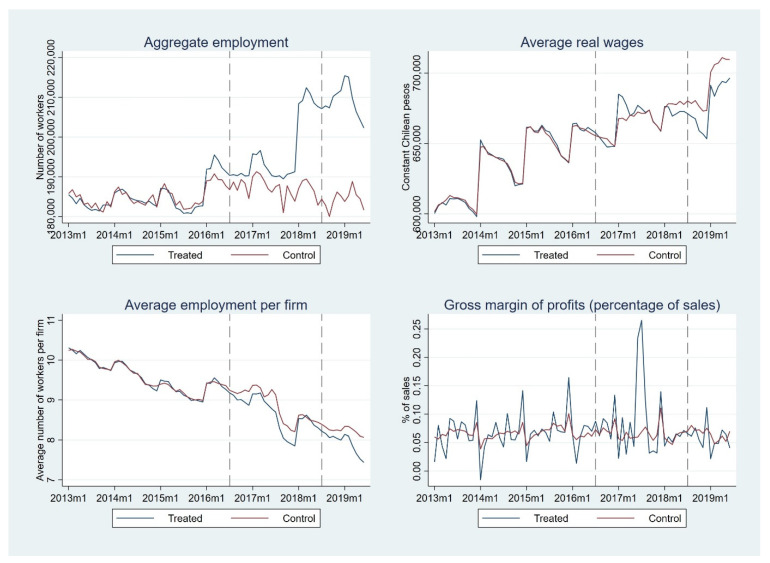
Aggregate employment, real wages, average workers per firm, and gross margin of profits in food and beverage industries, January 2013–June 2019.

Figure 2 shows the evolution for the dependent variables and their control groups. Synthetic control groups are formed with classes of economic activities from the non-metallic manufacturing sector in the case of the aggregate employment, the average number of workers per firm, and the gross margin of profits (as a proportion of sales). In the case of average real wages, it was not possible to form a suitable synthetic control group from the non-metallic sector, and classes of economic activities from the metallic sector and non-metallic sector (excluding the food and beverage sector) were considered. The specific classes that were used and their weights are not reported but are available from the authors upon request. Employment and wage data have strong seasonal effects, especially at the beginning of the year when there is a jump in figures. This can be explained by the filling of such information during April of each year. At that time, firms inform figures of employment and wages for the previous year, and it may happen that numbers informed for January of the previous year are significantly different than those informed a year before for the corresponding year (see top left graph in Figure 2). In any case, if this pattern is present not only for food and beverage manufacturing firms but also for other manufacturing firms, its effect should be controlled when considering the control groups.

Table 2 shows the results for the ITSA models. Non-significant parameters $\beta_{4}$ and $\beta_{5}$ shows that synthetic control groups are suitable in all cases as there are no statistical differences neither in levels nor in trends between them and treated groups in the pre-intervention period. Similarly, non-significant parameters $\beta_{6}$ and $\beta_{7}$ imply that after the implementation of the first stage of the FOP regulations, there were no changes in either levels or trends in the average number of workers per firm, the gross margin of profits, and the average real wages. In the case of aggregate employment (Column (1)), the ITSA shows a statistically significant increase in the trend after the implementation of the first stage of FOP regulations (i.e., aggregate employment increased at a monthly rate of 0.43% after the first stage of FOP regulations when compared to the synthetic control group, as shown by parameter $\beta_{7}$).

Regarding the second stage in the implementation of FOP regulations versus results for the first stage, parameters $\beta_{10}$ and $\beta_{11}$ mostly show no effect when comparing treated and control groups. In the case of real wages (Column (2)) and the gross profit margin (Column (4)), there is no effect in levels or in trends, while for aggregate employment (Column (1)) and average employment per firm (Column (3)), there is a small but statistically significant effect on the trends (parameter $\beta_{11}$). Concretely, aggregate employment decreases at a monthly rate of 0.68% after June 2018, when compared to the control group (as shown by parameter $\beta_{11}$ in Column (2)). In the case of average employment per firm, such a decrease is 0.59% (as shown by parameter $\beta_{11}$ in Column (3)).

**Figure A1:**
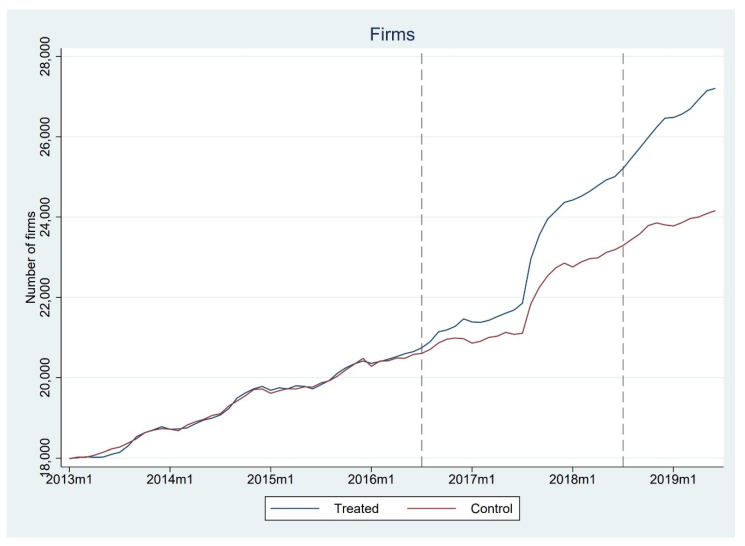
Evolution in the number of firms after two stages of FOP regulations.

The number of firms in the food and beverage manufacturing sector suffered significant changes during the period (as shown in Column (1) in Appendix A, Table A3). The ITSA on that variable shows that when compared to the pre-intervention period, the trend in the number of firms increased at a statistically significant monthly rate of 0.3%, compared to the control group, after the first stage of the FOP regulations (see parameter $\beta_{7}$ in Column (1) in Appendix A, Table A3 and Appendix A, Figure A1). There are no changes in levels or in trends with the second intervention between treated and control groups when comparing these with those for the first intervention period (see parameters $\beta_{10}$ and $\beta_{11}$ in Column (1) in Appendix A, Table A3, respectively).

Table 3 shows the results of the models when considering only one FOP regulation intervention (July 2016). In this case, results for parameter $\beta_{6}$ show that there was no statistically significant change in levels when comparing control and treated groups after the intervention. On the other hand, results for parameter $\beta_{7}$ are positive and statistically significant for aggregate employment (*p* < 0.01), negative for average real wages (*p* < 0.05), and gross margin of profits (*p* < 0.05). In the first case, the parameter shows a monthly increase of 0.4% for the aggregate employment for the treatment with respect to the control group, after the intervention (Column (1)). In the case of wages and the gross margin of profits, there is a monthly decrease of 0.1% for the treatment with respect to the control group, after the intervention (Columns (2) and (4), respectively).

## 4. Discussion

Using synthetic control groups to compare the evolution of classes of economic activities affected by the FOP regulations with others non-affected by them provides the opportunity to use a counterfactual statistic to enquire about what would have been the evolution of the dependent variables (aggregate employment, real wages, gross margin of profits, etc.) if such regulations would not have been implemented. Control groups constructed and used in this manuscript are suitable to that end.

Employment in the food and beverage manufacturing sector shows an increase in trend after the first phase of the Chilean law (when compared to the pre-intervention period and the control group, as shown by parameter $\beta_{7}$ in Table 2), which is partially reversed with the second phase (when compared to the first stage and the control group, as shown by parameter $\beta_{11}$ in Table 2). Overall, there is an increase in the trend of employment in this sector when compared to sectors used as controls. Average size of firms (in terms of employment) remained unchanged after FOP regulations, though after the second stage, there is a trend towards smaller firms. This indicates that the number of firms in the food and beverage manufacturing sector increased rapidly (vis-à-vis the control group) in a context of an increase in aggregate employment in such a sector (vis-à-vis the control group).

Real wages and gross margin of profits in food and beverage manufacturing sectors show neither a significant change after the first stage (when compared to the pre-intervention period and the control group, as shown by parameter $\beta_{7}$ in Table 2) nor after the second one (when compared to the first stage and the control group, as shown by parameter $\beta_{11}$ in Table 2). Overall, the FOP regulations meant a very small (and marginally significant) decrease in trends of wages and gross margin of profits (as shown by parameter $\beta_{7}$ in Table 3). If real wages and profits in food and beverage manufacturing sectors would have followed the same trajectory than those in control groups, they would have been 2.4% higher in real terms at the end of the three and half considered period (May 2019). This would have implied an economically negligible 0.8% higher real wage per year.

A previous study showed that after the first stage of FOP regulations in Chile, there was no statistically significant change, neither in aggregate employment nor in real wages in likely affected food and beverage manufacturing sectors [9]. In such a study, the control groups to compare with were food and beverage manufacturing sectors unlikely to be affected by those regulations. Results in this study assess the effect of the second stage of FOP regulations, with tighter limits of nutrients and increased numbers of products with warning labels and also by considering the effect of regulations not only on aggregate employment and real wages, but also on average employment per firm, and gross margin of profits; and, by considering the entire food and beverage manufacturing sector as the treated group and constructing synthetic control groups with non-food and beverage manufacturing sectors.

These results dispel the usual argument, proclaimed by the food and beverage industry and some think-tanks, that regulatory policies, such as taxes and/or labeling, have a negative effect on labor market outcomes [10,11,12,21]. Previous studies conducted in Chile, Mexico, and the US have shown the same lack of decrease in employment in the context of strong reductions in consumption of regulated products [9,22,23,24,25,26,27]. In the case of Chile, for instance, the reduction in SSB consumption was 23.7% after 18 months of the implementation of FOP regulations [26]; while breakfast cereals experienced a reduction of 26.4% in a comparable period [28].

As noted earlier, the Chilean set of regulations had significant changes in product reformulation [7] and food and beverage purchases of ‘high-in’ products [6]. We noted earlier the significant changes in marketing and exposure to children. There is no study yet on the evolution of prices for “high-in” and not-high-in products, but it may be the case that firms would decrease the prices of the less desirable products (“high-in” product) and increase those of their more demanded substitutes (not-high-in products) to maintain profit margins.

Overall, labor market outcomes and profits were largely unaffected by these regulations. These could be due to firms producing both “high-in” and not-high-in substitutes (e.g., firms producing SSBs and bottled water); and/or reformulation of products to convert them into not-high-in ones, which imply maintaining the demand faced by firms. While the first alternative would eventually imply reallocating workers across lines of production, the second one would mean investing in research and development to change formulas and market the reformulated products. Though with the existing data we cannot know the economic effects of both alternatives, such data show that in any case, there were neither significant effects on labor market outcomes nor on aggregate gross margin of profits.

There is no surprise in these results from both an empirical and a theoretical perspective. When households decide not to spend on high-in products, they could save it or spend it on other products. It is highly unlikely that households would increase savings because of food and beverage labeling regulations, especially in a context of increasing household indebtedness (household non-mortgage debt went from 18.4% of the gross domestic product in 2015 to 19.5% in 2017) [29]. Though there is no information yet on how households changed their budget after the FOP regulations, it is likely that the percentage spent on food and beverage remained relatively constant and, consequently, there was a reallocation of budget from “high-in” products to not-high-in products.

The analyses conducted here have limitations that need to be made explicit. Firstly, we do not have information at the firm level, but at the level of class of economic activity. This may hide the effects that FOP regulations had on specific firms and/or on specific sectors of the food and beverage manufactures. However, even if we have such information, it would be almost impossible to attribute effects in the case of firms producing both “high-in” and not-high-in products. We have no information on the proportion of firms that produce both types of products.

Secondly, we would ideally have also evaluated the period starting in June 2019 for phase 3. However, two sets of events meant that from October 2019 until July 2020, there was major economic disruption due to massive demonstrations in all cities in the country and then COVID-19, which led to a complete shutdown of most commercial activity. With too few months of activity in phase 3 (July through September 2019), we do not have the ability to evaluate the impact on phase 3.

## 5. Conclusions

Chilean FOP regulations have been assessed as the “the world’s most ambitious comprehensive attempt to remake a country’s food culture” [30,31]. Such regulations have been associated, at least, with significant decreases in the intake of critical nutrients and to an important reformulation of products to avoid the FOP warning labels and related regulations [6]. These positive changes were not achieved at the cost of less employment, lower real wages, or less gross margin of profits for the food and beverage sector. The results presented here suggest that after the first two stages of implementation of the FOP regulations, there were no economically significant changes in these variables. The experience of Chile suggests that if similar regulations were adopted elsewhere, they would have similar results, especially considering that the food and beverage sector in most countries is dominated by the same multinational companies, with similar product portfolio, and comparable production processes [32,33].

## Figures and Tables

**Table 1:** Descriptive statistics for aggregate employment (number of workers), average real wages (in constant Chilean pesos), average number of workers (number of workers per firm), and gross profit margins (as proportion of sales).

		Aggregate Employment (Workers)		Average Real Wages (Constant Pesos)		Gross Margin of Profits (as Proportion of Sales)		Average Employment Per Firm (Workers Per Firm)	
		Treated	Control	Treated	Control	Treated	Control	Treated	Control
Pre-intervention (January 2013–May 2016)	Mean	184,971	184,867	635,964	635,864	0.053	0.053	9.60	9.60
	Std. Dev.	3739	2462	22,425	21,292	0.035	0.017	0.39	0.38
First intervention (July 2016–May 2018)	Mean	196,067	187,304	667,339	666,658	0.074	0.053	8.70	8.87
	Std. Dev.	8200	2458	11,274	9827	0.069	0.021	0.43	0.41
Change (%)	Mean	6.0	1.3	4.9	4.8	39.6	0.0		
Second intervention (July 2018–May 2019)	Mean	209,663	184,576	675,431	690,590	0.047	0.052	7.98	8.26
	Std. Dev.	3514	2171	15,606	15,989	0.024	0.017	0.22	0.08

**Table 2:** Changes in labor market and firms’ profit outcomes after two stages of FOP regulations.

	Aggregate Employment	Average Real Wages	Average Employment	Gross Margin of Profits
VARIABLES	(1)	(2)	(3)	(4)
$\mathrm{Trend}\;\mathrm{of}\;\mathrm{dependent}\;\mathrm{variable}\;\mathrm{pre-intervention}\;(\beta_{1}$)	−0.0003	0.0030 ***	−0.0032 ***	0.0011
	(0.0004)	(0.0003)	(0.0004)	(0.0007)
Difference in levels of dependent variable for	−0.0068	−0.0020	0.0010	−0.0064
$\mathrm{groups}\;\mathrm{pre-intervention}\;(\beta_{4}$)	(0.0051)	(0.0088)	(0.0071)	(0.0066)
Difference in trends of dependent variable for	0.0004	0.0001	−0.0001	0.0003
$\mathrm{groups}\;\mathrm{pre-intervention}\;(\beta_{5}$)	(0.0004)	(0.0004)	(0.0004)	(0.0003)
Change in level of dependent variable after first	0.0229 ***	−0.0196 ***	0.0597 ***	−0.0093
$\mathrm{intervention}\;(\beta_{2}$)	(0.0060)	(0.0064)	(0.0128)	(0.0084)
Change in trend of dependent variable after first	−0.0018 ***	−0.0008 **	−0.0038 ***	0.0003
$\mathrm{intervention}\;(\beta_{3}$)	(0.0003)	(0.0004)	(0.0007)	(0.0005)
Difference in level of dependent variable for groups	−0.0169	0.0047	−0.0253	0.0159
$\mathrm{after}\;\mathrm{first}\;\mathrm{intervention}\;(\beta_{6}$)	(0.0146)	(0.0101)	(0.0166)	(0.0156)
Difference in trends of dependent variable for	0.0043 ***	−0.0006	0.0003	−0.0008
$\mathrm{groups}\;\mathrm{after}\;\mathrm{first}\;\mathrm{intervention}\;(\beta_{7}$)	(0.0011)	(0.0006)	(0.0010)	(0.0008)
Change in level of dependent variable after second	0.0077	−0.0013	0.0428 ***	0.0001
$\mathrm{intervention}\;(\beta_{8}$)	(0.0066)	(0.0049)	(0.0118)	(0.0093)
Change in trend of dependent variable after second	−0.0004	0.0027 ***	−0.0000	−0.0004
$\mathrm{intervention}\;(\beta_{9}$)	(0.0005)	(0.0004)	(0.0009)	(0.0009)
Difference in level of dependent variable for groups	0.0341	−0.0129 *	0.0152	−0.0196
$\mathrm{after}\;\mathrm{second}\;\mathrm{intervention}\;(\beta_{10}$)	(0.0220)	(0.0070)	(0.0231)	(0.0170)
Difference in trends of dependent variable for	−0.0068 ***	−0.0001	−0.0059 ***	0.0009
$\mathrm{groups}\;\mathrm{after}\;\sec\mathrm{ond}\;\mathrm{intervention}\;(\beta_{11}$)	(0.0016)	(0.0006)	(0.0019)	(0.0015)
Logarithm non-mining IMACEC	0.3976 **	−0.3566 ***	0.2619	−0.5461
	(0.1682)	(0.1026)	(0.1614)	(0.3487)
Constant	10.3198 ***	14.9560 ***	1.1371	2.5279
	(0.7663)	(0.4706)	(0.7355)	(1.5885)
Observations	156	156	156	156

*** *p* < 0.01, ** *p* < 0.05, * *p* < 0.1. Standard errors in parentheses.

**Table 3:** Changes in labor market and firms’ profit outcomes considering one FOP regulation intervention (July 2016).

Variables	Aggregate Employment (1)	Average Real Wages (2)	Average Employment (3)	Gross Margin of Profits (4)
$\mathrm{Trend}\;\mathrm{of}\;\mathrm{dependent}\;\mathrm{variable}\;\mathrm{pre-intervention}\;(\beta_{1}$)	−0.0002	0.0030 ***	−0.0030 ***	0.0010
	(0.0004)	(0.0003)	(0.0004)	(0.0006)
$\mathrm{Difference}\;\mathrm{in}\;\mathrm{levels}\;\mathrm{of}\;\mathrm{dependent}\;\mathrm{variable}\;\mathrm{for}\;\mathrm{groups}\;\mathrm{pre-intervention}\;(\beta_{4}$)	−0.0068	−0.0020	0.0010	−0.0064
	(0.0048)	(0.0088)	(0.0064)	(0.0065)
$\mathrm{Difference}\;\mathrm{in}\;\mathrm{trends}\;\mathrm{of}\;\mathrm{dependent}\;\mathrm{variable}\;\mathrm{for}\;\mathrm{groups}\;\mathrm{pre-intervention}\;(\beta_{5}$)	0.0004	0.0001	−0.0001	0.0003
	(0.0004)	(0.0004)	(0.0004)	(0.0003)
$\mathrm{Change}\;\mathrm{in}\;\mathrm{level}\;\mathrm{of}\;\mathrm{dependent}\;\mathrm{variable}\;\mathrm{after}\;\mathrm{first}\;\mathrm{intervention}\;(\beta_{2}$)	0.0192 ***	−0.0247 ***	0.0422 ***	−0.0076
	(0.0060)	(0.0075)	(0.0124)	(0.0071)
$\mathrm{Change}\;\mathrm{in}\;\mathrm{trend}\;\mathrm{of}\;\mathrm{dependent}\;\mathrm{variable}\;\mathrm{after}\;\mathrm{first}\;\mathrm{intervention}\;(\beta_{3}$)	−0.0015 ***	−0.0003	−0.0020 ***	0.0001
	(0.0002)	(0.0003)	(0.0004)	(0.0003)
$\mathrm{Difference}\;\mathrm{in}\;\mathrm{level}\;\mathrm{of}\;\mathrm{dependent}\;\mathrm{variable}\;\mathrm{for}\;\mathrm{groups}\;\mathrm{after}\;\mathrm{first}\;\mathrm{intervention}\;(\beta_{6}$)	−0.0114	0.0090	−0.0161	0.0199
	(0.0147)	(0.0105)	(0.0178)	(0.0182)
$\mathrm{Difference}\;\mathrm{in}\;\mathrm{trends}\;\mathrm{of}\;\mathrm{dependent}\;\mathrm{variable}\;\mathrm{for}\;\mathrm{groups}\;\mathrm{after}\;\mathrm{first}\;\mathrm{intervention}\;(\beta_{7}$)	0.0039 ***	−0.0011 **	−0.0006	−0.0013 **
	(0.0007)	(0.0005)	(0.0007)	(0.0006)
Logarithm non-mining IMACEC	0.3439 **	−0.3399 ***	0.1518	−0.5249
	(0.1679)	(0.1132)	(0.1620)	(0.3271)
Constant	10.5644 ***	14.88 ***	1.6384 **	2.4315 *
	(0.7648)	(0.5183)	(0.7380)	(1.4904)
Observations	156	156	156	156

*** *p* < 0.01, ** *p* < 0.05, * *p* < 0.1. Standard errors in parentheses.

## Data Availability

The Central Bank tax data is unavailable for use outside of the Bank and its use must be approved by the team at the Central Bank. Chile also has aggregate employment data by sector publicly available.

## Appendix A

**Table A1:** Sequence of implementation and limits for FOP warning labels.

Table 1.	Nutrient or Energy	Phase 1: 26 June 2016	Phase 2: 24 Months after Beginning of Phase 1 (27 June 2018)	Phase 3: 36 Months after Beginning of Phase 1 (27 June 2019)
Solid foods	Energy, kilocalories (kcal)/100 g (g)	350.0	300.0	275.0
	Sodium, milligrams (mg)/100 g	800.0	500.0	400.0
	Total sugars, g/100 g	22.5	15.0	10.0
	Saturated fats, g/100 g	6.0	5.0	4.0
Liquid foods	Energy, kcal/100 milliliters (mL)	100.0	80.0	70.0
	Sodium, mg/100 mL	100.0	100.0	100.0
	Total sugars, g/100 mL	6.0	5.0	5.0
	Saturated fats, g/100 mL	3.0	3.0	3.0

Source: Based on https://www.minsal.cl/ley-de-alimentos-nuevo-etiquetado-de-alimentos/. Accessed on 9 November 2021.

**Table A2:** Classification of food and beverage manufacturing industries.

Classification (ISIC Revision 4 code and Spanish translation in parenthesis)
Solid food industries
Slaughterhouse operation (10101 Explotación de mataderos)
Production of fishmeal (10201 Producción de harina de pescado)
Processing and preserving of meat (10102 Elaboración y conservación de carne y productos cárnicos)
Production of fish meal (10201 Producción de harina de pescado)
Processing and preserving of salmonids (10202 Elaboración y conservación de salmónidos)
Processing and preserving of other fish (in land) (10203 Elaboración y conservación de otros pescados, en plantas situadas en tierra (excepto barcos de factoría)
Processing and preserving of crustaceans and molluscs (10204 Elaboración y conservación de crustáceos, moluscos, invertebrados acuáticos y otros productosacuáticos, en plantas situadas en tierra (excepto barcos factoría)
Processing and preserving of other fish (factory ships) (10205 Actividades de elaboración y conservación de pescado, realizadas en barcos factoría)
Production of food based on sea algae (10206 Elaboración y procesamiento de algas)
Processing and preserving of fruit and vegetables (10300 Elaboración y conservación de frutas, legumbres y hortalizas)
Manufacture of vegetable and animal oils and fats (10400 Elaboración de aceites y grasas de origen vegetal y animal)
Manufacture of dairy products (10205 Actividades de elaboración y conservación de pescado, realizadas en barcos factoría)
Manufacture of grain mill products (10610 Elaboración de productos de molinería)
Manufacture of starches and starch products (10620 Elaboración de almidones y productos derivados del almidón)
Manufacture of bakery products (10710 Elaboración de productos de panadería)
Manufacture of sugar (10720 Elaboración de azúcar)
Manufacture of cocoa, chocolate and sugar confectionery (10730 Elaboración de cacao, chocolate y de productos de confitería)
Manufacture of macaroni, noodles, couscous and similar farinaceous products (10740 Elaboración de macarrones, fideos, alcuzcuz y productos farináceos similares)
Manufacture of prepared meals and dishes (10750 Elaboración de comidas y platos preparados)
Manufacture of other food products n.e.c. (10790 Elaboración de otros productos alimenticios n.c.p.)
Beverage industries
Distilling, rectifying and blending of pisco (11011 Elaboración de pisco (industrias pisqueras))
Distilling, rectifying and blending of spirits, exept pisco (11012 Destilación, rectificación y mezclas de bebidas alcohólicas; excepto pisco)
Manufacture of wines (11020 Elaboración de vinos)
Manufacture of malt liquors and malt (11030 Elaboración de bebidas malteadas y de malta)
Manufacture of soft drinks; production of mineral waters and other bottled waters (11040 Elaboración de bebidas no alcohólicas; producción de aguas minerales y otras aguas embotelladas)

**Table A3:** Changes in the number of firms and the gross margin of profit (including expenditures on capital goods) after two stages of FOP regulations.

Variables	Number of Firms (1)	Gross Margin of Profits (2)
	0.0029 ***	0.0015 *
$\mathrm{Trend}\;\mathrm{of}\;\mathrm{dependent}\;\mathrm{variable}\;\mathrm{pre}\text{-}\;\mathrm{intervention}\;(\beta_{1}$)	(0.0003)	(0.0008)
	−0.0025	−0.0049
$\mathrm{Difference}\;\mathrm{in}\;\mathrm{levels}\;\mathrm{of}\;\mathrm{dependent}\;\mathrm{variable}\;\mathrm{for}\;\mathrm{groups}\;\mathrm{pre}\text{-}\mathrm{intervention}\;(\beta_{4}$)	(0.0034)	(0.0069)
	0.0001	0.0002
$\mathrm{Difference}\;\mathrm{in}\;\mathrm{trends}\;\mathrm{of}\;\mathrm{dependent}\;\mathrm{variable}\;\mathrm{for}\;\mathrm{groups}\;\mathrm{pre}\text{-}\mathrm{intervention}\;(\beta_{5}$)	(0.0001)	(0.0003)
	−0.0230 ***	−0.0088
$\mathrm{Change}\;\mathrm{in}\;\mathrm{level}\;\mathrm{of}\;\mathrm{dependent}\;\mathrm{variable}\;\mathrm{after}\;\mathrm{first}\;\mathrm{intervention}\;(\beta_{2}$)	(0.0075)	(0.0097)
	0.0025 ***	−0.0003
$\mathrm{Change}\;\mathrm{in}\;\mathrm{trend}\;\mathrm{of}\;\mathrm{dependent}\;\mathrm{variable}\;\mathrm{after}\;\mathrm{first}\;\mathrm{intervention}\;(\beta_{3}$)	(0.0005)	(0.0006)
	−0.0015	0.0142
$\mathrm{Difference}\;\mathrm{in}\;\mathrm{level}\;\mathrm{of}\;\mathrm{dependent}\;\mathrm{variable}\;\mathrm{for}\;\mathrm{groups}\;\mathrm{after}\;\mathrm{first}\;\mathrm{intervention}\;(\beta_{6}$)	(0.0125)	(0.0162)
	0.0033 ***	−0.0003
$\mathrm{Difference}\;\mathrm{in}\;\mathrm{trends}\;\mathrm{of}\;\mathrm{dependent}\;\mathrm{variable}\;\mathrm{for}\;\mathrm{groups}\;\mathrm{after}\;\mathrm{first}\;\mathrm{intervention}\;(\beta_{7}$)	(0.0008)	(0.0008)
$\mathrm{Change}\;\mathrm{in}\;\mathrm{level}\;\mathrm{of}\;\mathrm{dependent}\;\mathrm{variable}\;\mathrm{after}\;\sec\mathrm{ond}\;\mathrm{intervention}\;(\beta_{8}$)	−0.0063	0.0070
	(0.0062)	(0.0115)
$\mathrm{Change}\;\mathrm{in}\;\mathrm{trend}\;\mathrm{of}\;\mathrm{dependent}\;\mathrm{variable}\;\mathrm{after}\;\sec\mathrm{ond}\;\mathrm{intervention}\;(\beta_{9}$)	−0.0025 ***	−0.0002
	(0.0007)	(0.0014)
$\mathrm{Difference}\;\mathrm{in}\;\mathrm{level}\;\mathrm{of}\;\mathrm{dependent}\;\mathrm{variable}\;\mathrm{for}\;\mathrm{groups}\;\mathrm{after}\;\sec\mathrm{ond}\;\mathrm{intervention}\;(\beta_{10}$)	−0.0045	−0.0301
	(0.0095)	(0.0192)
$\mathrm{Difference}\;\mathrm{in}\;\mathrm{trends}\;\mathrm{of}\;\mathrm{dependent}\;\mathrm{variable}\;\mathrm{for}\;\mathrm{groups}\;\mathrm{after}\;\sec\mathrm{ond}\;\mathrm{intervention}\;(\beta_{11}$)	0.0006	0.0010
	(0.0009)	(0.0019)
Logarithm non-mining IMACEC	0.3147 **	−0.6631 *
	(0.1432)	(0.3988)
Constant	8.3632 ***	3.0357 *
	(0.6528)	(1.8170)
Difference between the treatment and control groups in comparing trends of the pre.intervention and the second intervention period (β7 + β11)	0.0037 ***	0.0007
	(0.0006)	(0.0017)
Observations	156	156

*** *p* < 0.01, ** *p* < 0.05, * *p* < 0.1. Standard errors in parentheses.
